# Assessment of Hip Abduction Motion Assistance Using a Single-Joint Hybrid Assistive Limb Robot: Feasibility and Safety Evaluation in Healthy Adults

**DOI:** 10.3390/jcm14020454

**Published:** 2025-01-12

**Authors:** Fumi Hirose, Tomofumi Nishino, Yukiyo Shimizu, Yuichiro Soma, Ayumu Haginoya, Shota Yasunaga, Koshiro Shimasaki, Ryunosuke Watanabe, Tomohiro Yoshizawa, Hajime Mishima

**Affiliations:** 1 Department of Orthopaedic Surgery, Institute of Medicine, University of Tsukuba, Tsukuba 305-8575, Japan; f.ochiai.0023@tsukuba-seikei.jp (F.H.); tksoma0720@yahoo.co.jp (Y.S.); syasunaga@tsukuba-seikei.jp (S.Y.); koshiro19881020@tsukuba-seikei.jp (K.S.); ryuwatanabe@tsukuba-seikei.jp (R.W.); tyoshizawa@tsukuba-seikei.jp (T.Y.); hmishima@tsukuba-seikei.jp (H.M.); 2 Department of Rehabilitation Medicine, Institute of Medicine, University of Tsukuba, Tsukuba 305-8575, Japan; shimiyukig@md.tsukuba.ac.jp; 3 Department of Rehabilitation Medicine, University of Tsukuba Hospital, Tsukuba 305-8576, Japan; ayumu_haginoya@yahoo.co.jp

**Keywords:** hybrid assistive limb robot, hip joint abduction, gluteus medius, flexion angle, maximum voluntary contraction

## Abstract

**Background/Objectives:** Preoperative muscle atrophy leads to persistent gait abnormalities in patients undergoing total hip arthroplasty (THA). Efficient motor learning of the gluteus medius is crucial for their recovery. In this study, a single-joint hybrid assistive limb (HAL) was developed to assist hip abduction. We aimed to evaluate the muscle activity and safety of this device during hip abduction in healthy adults. **Methods:** Ten healthy adults (five males and five females; mean age, 40.7 years) with no hip disorders performed one set of 30 repetitions of side-lying hip abduction under three conditions: without HAL (pre-HAL), with HAL, and without HAL (post-HAL). Muscle activities of the gluteus medius, gluteus maximus, tensor fasciae latae, rectus femoris, and biceps femoris (expressed as percentage of maximum voluntary contraction [%MVC]); vital signs; hip visual analog scale (VAS); and hip abduction and flexion angles were assessed. The mean values were compared among the conditions. **Results:** The %MVC of the gluteus medius significantly increased from 52% (pre-HAL) to 75.4% (HAL) and then decreased slightly to 61.6% (post-HAL). No other muscle groups showed significant changes. Vital signs and hip VAS scores showed no significant variation. Although no significant differences were found in the hip abduction and flexion angles, a reduction in the hip flexion angle was observed in the HAL and post-HAL conditions. **Conclusions:** The hip abduction HAL effectively and safely enhanced gluteus medius activity. Reduction in the hip flexion angle during HAL and post-HAL suggests the possibility of appropriate abduction movements and motor learning effects.

## 1. Introduction

Hip osteoarthritis is characterized by pain and a reduced range of motion, leading to limitations in gait function and activities of daily living (ADL). Specific gait impairments include reduced stride length and walking speed and abnormal gait patterns.

Total hip arthroplasty (THA) is a common surgical treatment for hip osteoarthritis [1] and is recognized as an effective method for alleviating pain and improving ADL [2]. Improvements in contralateral hip and knee joint range of motion, single-leg support time symmetry, stride length, and walking speed have been reported for up to 12 months after THA [3]. However, residual gait abnormalities may persist after THA because of preoperative muscle atrophy or contracture of the periarticular hip muscles [4,5]. Among these abnormalities, Trendelenburg gait is a representative feature in patients with hip osteoarthritis. This gait pattern is characterized by pelvic tilt during the stance phase and is attributed to weakness or dysfunction of the hip abductor muscles, particularly the gluteus medius [6]. Furthermore, Trendelenburg gait is recognized as one of the postoperative complications following THA [7]. Previous studies have reported that its incidence varies depending on the surgical approach employed, likely due to differences in the extent of soft tissue dissection and potential damage to the abductor musculature [8]. Patients with hip osteoarthritis are concerned about gait abnormalities before and after surgery [9], making gait improvement a primary treatment goal.

Restoring a stable gait requires functional recovery of the hip abductor muscle group, particularly the gluteus medius. Conventional rehabilitation programs commonly include strengthening exercises using TheraBands or weights, as well as passive and active range-of-motion exercises and manual therapy. However, when muscle weakness is severe or incorrect movement patterns have persisted for a prolonged period, acquiring a proper movement pattern can be difficult. As a result, efficient motor learning may be hindered, highlighting the need for more targeted interventions that address both muscular strength and neuromuscular control.

A decrease in hip abductor muscle strength results in gait abnormalities such as the Trendelenburg gait, emphasizing the critical role of the hip abductor muscles in achieving stable walking. The muscles responsible for hip abduction include the gluteus medius, gluteus minimus, and tensor fasciae latae; the gluteus medius is particularly important. Given the importance of the gluteus medius in achieving a stable gait, we hypothesized that efficient motor learning targeting the gluteus medius post-THA could improve gait patterns. However, it is difficult to efficiently perform hip abduction training using the existing devices. We consider the hybrid assistive limb (HAL) system to be a promising tool for facilitating motor learning, specifically aimed at improving the activation and function of the hip abductors, particularly the gluteus medius. Previous studies have reported that using HAL for gait training in the postoperative rehabilitation of patients undergoing THA leads to improvements in hip extension angles and gait parameters [10]. However, to our knowledge, no prior study has focused on using HAL specifically to improve the function of the hip abductor muscles, including the gluteus medius.

HAL is an exoskeletal robot that controls and assists movements based on bioelectrical activity generated through voluntary movements. It has been hypothesized that by generating feedback from the central nervous system, this device stimulates functional recovery by inducing plasticity in the impaired central nervous system [11]. As the single-joint HAL is designed to support uniaxial movements, such as flexion and extension of the knee or elbow joints, it alone cannot facilitate hip abduction movements. At our institution, we previously developed and evaluated the safety and efficacy of modified single-joint HAL systems, such as the shoulder HAL for flexion and extension of the shoulder joint [12] and the ankle HAL for dorsiflexion and plantarflexion of the ankle joint [13]. Based on this experience, we adapted the shoulder HAL attachment to enable a single-joint HAL to perform hip abduction movements.

This study aimed to evaluate the effectiveness and safety of the single-joint HAL in assisting the hip abduction muscles during hip abduction movements in healthy adults.

## 2. Materials and Methods

### 2.1. Participants

Ten healthy adults participated in our study. None of the participants had received active treatment for circulatory or respiratory conditions at the time of testing. None had a history of hip trauma or surgery. Participants were excluded if they met any of the following criteria: body size incompatible with HAL specifications (applicable range: height, 150–190 cm; weight, 40–100 kg), inability to attach HAL bioelectrodes due to skin conditions, inability to provide informed consent, or any condition deemed unsuitable by the attending physician.

This study was conducted in accordance with the Declaration of Helsinki and was approved by the Ethics Committee of Tsukuba University Faculty of Medicine (approval no.: R05-103, approval date: 20 September 2023). This study was registered with the Japan Registry of Clinical Trials Registry (jRCT1032230385). All patients provided written informed consent for participation and publication, including the use of the accompanying images.

### 2.2. Setup of the Single-Joint HAL

**Figure 1:**
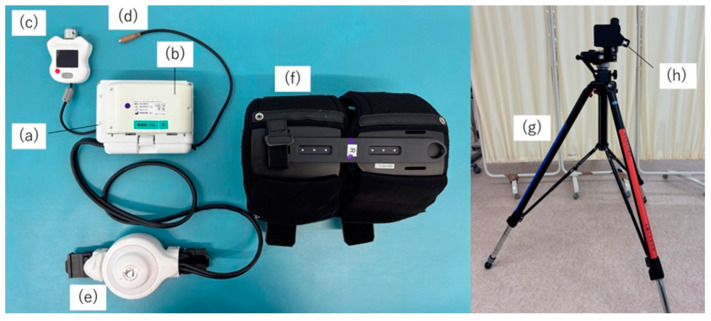
Structure of the hip abduction HAL. The hip abduction HAL system consists of (**a**) a control device, (**b**) a battery, (**c**) a manual controller, (**d**) an electrode cable, (**e**) a power unit, (**f**) a thigh belt, (**g**) a tripod-based platform, and (**h**) an attachment. HAL, hybrid assistive limb robot.

The hip abduction HAL system consists of a control box, battery, power unit, attachment, controller, electrode cables, thigh belt, and a tripod-based platform with attachments (Figure 1).

For the single-joint HAL targeting the knee, the lower leg component was secured to a tripod-based platform with attachments, and the thigh component was similarly fastened to the participant’s thigh. To attach the unit to the platform, we employed a specialized attachment originally designed and manufactured for a previously developed shoulder HAL device. For thigh fixation, we used a standard thigh fixation belt with the HAL system, without modification.

**Figure 2:**
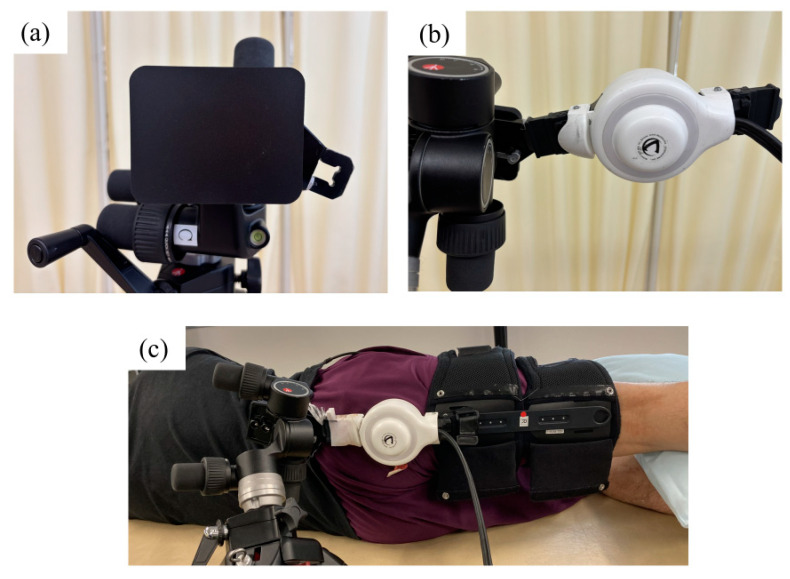
(**a**,**b**) The distal portion of the single-joint HAL, originally designed for the knee joint, is attached to the tripod-based platform using a custom attachment. (**c**) The proximal portion is secured to the participant’s thigh using the standard thigh belt. Participants are positioned in a side-lying posture on a bed near the tripod-based platform. The rotation axis of the single-joint HAL is aligned with the femoral axis, and the power unit is adjusted to rest at the level of the greater trochanter against the gluteal region. HAL, hybrid assistive limb robot.

The participants were positioned in a side-lying posture on a bed near a tripod-based platform. The rotation axis of the single-joint HAL was aligned with the femoral axis, and the power unit was adjusted to rest at the level of the greater trochanter against the gluteal region (Figure 2). To detect the bioelectrical signals during hip abduction, sensors were placed on the surface of the gluteus medius. The electrodes were placed based on anatomical landmarks following the SENIAM guidelines [14], positioned along the line connecting the iliac crest and greater trochanter at 50% of the line’s length. Proper placement was confirmed by detecting muscle activity in the gluteus medius during isometric contractions in voluntary hip abduction movements.

### 2.3. Hip Abduction–Adduction Movement

The participants were instructed to perform a cycle of right hip abduction and adduction in a left-side-lying position at a speed of 60 cycles per minute; the timing was ensured using a metronome. They were also instructed to maintain 0° hip flexion throughout the exercise. Abduction movements were performed within the range of the neutral position to the participant’s maximum achievable abduction angle. Each set consisted of 30 abduction–adduction cycles.

The exercises were conducted in the following sequence: hip abduction–adduction without HAL assistance (pre-HAL), with HAL assistance (HAL), and without HAL assistance (post-HAL), with one set performed under each condition. One reason for setting the intervention to 30 repetitions is that our standard rehabilitation protocol typically involves a total of 30 assisted hip abduction exercises. Since our participants were healthy individuals, we chose one continuous set of 30 repetitions. We also determined that 30 repetitions would provide sufficient muscle activity while avoiding excessive muscle fatigue. Throughout the intervention, we verbally confirmed whether the participants experienced any fatigue, ensuring that no decline in performance occurred due to excessive muscular fatigue.

### 2.4. Evaluation

#### 2.4.1. Muscle Activity

Muscle activity was recorded in the gluteus medius, gluteus maximus, tensor fasciae latae, rectus femoris, and biceps femoris muscles using a wireless surface electromyography (EMG) device (Ultium EMG; Noraxon, Scottsdale, AZ, USA).

**Figure 3:**
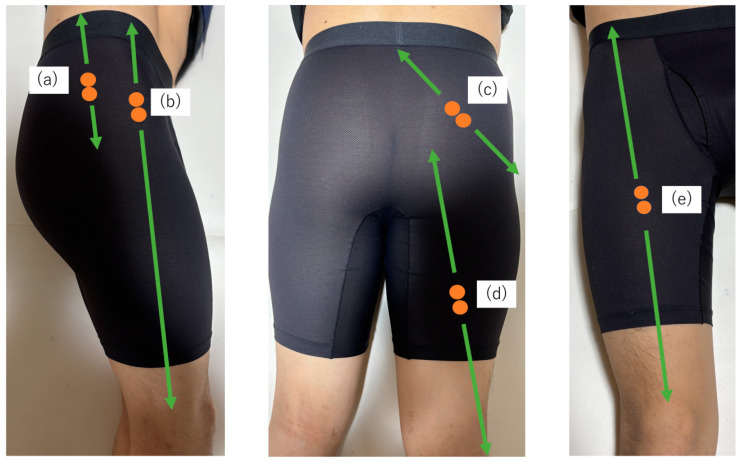
The EMG electrode placement: (**a**) gluteus medius, (**b**) tensor fasciae latae, (**c**) gluteus maximus, (**d**) biceps femoris, and (**e**) rectus femoris.

Bipolar Ag-AgCl surface electrodes, each measuring 1 cm in diameter and with a center-to-center distance of 2.5 cm were used. Before electrode application, the skin was wiped with alcohol to reduce impedance, and pairs of electrodes were attached. Pairs of surface electrodes were applied to the right lower limb on the gluteus medius, gluteus maximus, tensor fasciae latae, rectus femoris, and biceps femoris based on the SENIAM recommendations [12] (Figure 3):

▪ Gluteus medius: Positioned along the line connecting the iliac crest and greater trochanter at 50% of the line length.
▪ Gluteus maximus: Positioned along the line connecting the sacrum and greater trochanter at 50%, following the line connecting the posterior superior iliac spine and the midline of the posterior thigh.
▪ Tensor fasciae latae: Positioned along the line connecting the anterior superior iliac spine and lateral femoral condyle at the proximal one-sixth of the line length.
▪ Rectus femoris: Positioned along the line connecting the anterior superior iliac spine and the superior edge of the patella at 50% of the line length.
▪ Biceps femoris: Positioned along the line connecting the ischial tuberosity and lateral tibial condyle at 50% of the line length.

To standardize the EMG amplitude, the maximum voluntary contraction (MVC) of each muscle was used as a reference. Before the sessions, the MVC was measured for each muscle using 5 s maximal effort isometric contractions against manual resistance in the following positions:

▪ Gluteus medius and tensor fasciae latae: Side-lying position with the hip abducted.
▪ Gluteus maximus: Prone position with the hip extended.
▪ Rectus femoris: Sitting position with the knee extended.
▪ Biceps femoris: Prone position with the knee flexed.

After MVC measurements, EMG data were recorded during the pre-HAL, HAL, and post-HAL sessions.

The EMG data of each muscle obtained by the device were filtered using a bandpass filter of 20–500 Hz and then smoothed with a root mean square filter with a 100 ms window width. Smoothed EMG amplitudes were normalized to the peak values of the corresponding MVC waveforms.

During the abduction–adduction movements in each session, the EMG waveforms were analyzed for 30 cycles from the neutral to the maximum abduction position. The smoothed amplitude of the EMG waveform of each muscle was normalized as a percentage of the corresponding MVC (%MVC) and averaged across all cycles for each session.

#### 2.4.2. Motion Analysis

Hip abduction and flexion angles during hip abduction–adduction movements were recorded using a three-dimensional inertial sensor-based motion analysis system (Ultium Motion, Noraxon, Scottsdale, AZ, USA). Wireless sensors were placed on the sacrum, lateral mid-thigh, and lateral proximal shank. The hip abduction angle was defined as the angle between the limb and perpendicular axes relative to the limb. The hip flexion angle was defined as the angle between the body and femoral axes.

Muscle activity measured by the wireless surface EMG system was recorded in synchronization with motion capture for the analysis of muscle activation in accordance with the hip abduction–adduction movement.

For each session, the peak values of hip abduction and flexion angles were detected for 30 cycles from the neutral position to the maximum abduction position. Average peak values were calculated for each session.

#### 2.4.3. Safety

Vital signs, including blood pressure, heart rate, and blood oxygen saturation, were measured immediately before and after the pre-HAL, HAL, and post-HAL sessions. Hip pain was evaluated using a visual analog scale (VAS). The participants were asked about their pain levels at the same time points: before and after the pre-HAL, HAL, and post-HAL sessions.

### 2.5. Statistical Analysis

The mean values were calculated for all evaluation parameters. For %MVC and hip abduction and flexion angles, comparisons were made across the pre-HAL, HAL, and post-HAL conditions. Vital signs and hip pain, as assessed by the VAS, were compared before and after the pre-HAL, HAL, and post-HAL sessions.

Repeated-measures analysis of variance was performed for all comparisons. Post hoc analyses were conducted using Bonferroni’s multiple comparison tests, examining three pairwise conditions: pre-HAL vs. HAL, HAL vs. post-HAL, and pre-HAL vs. post-HAL. Differences were considered statistically significant at *p* < 0.05.

Analyses were conducted using IBM SPSS Statistics for Windows, Version 29 (Released 2021; IBM Corp., Armonk, NY, USA).

## 3. Results

### 3.1. Participants’ Demographic Characteristics

Ten individuals (five males and five females; mean age, 40.7 years) participated in this study. Their mean height was 1.66 m (range: 1.54–1.77 m), mean weight was 63.6 kg (range: 52–84 kg), and mean was BMI 23 (range: 19.6–27.1). All of these values fell within the operational range for the single-joint HAL device used in this study, confirming that the participants were adequately able to don and operate the device even when individual differences were taken into consideration.

### 3.2. Visual Assessment

**Figure 4:**
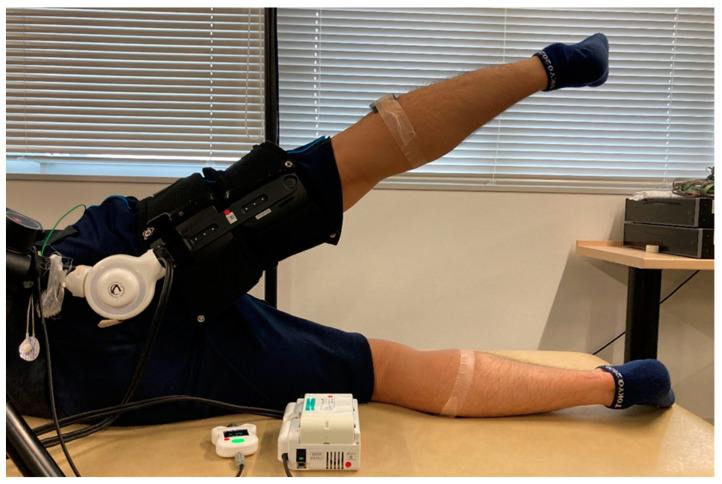
Images of the hip abduction HAL device. Participants are able to perform hip abduction–adduction movements continuously and smoothly while using the hip abduction HAL. HAL, hybrid assistive limb robot.

Visual assessment confirmed that hip abduction–adduction movements could be performed continuously and smoothly when using the hip abduction HAL (Figure 4).

### 3.3. Muscle Activity

**Figure 5:**
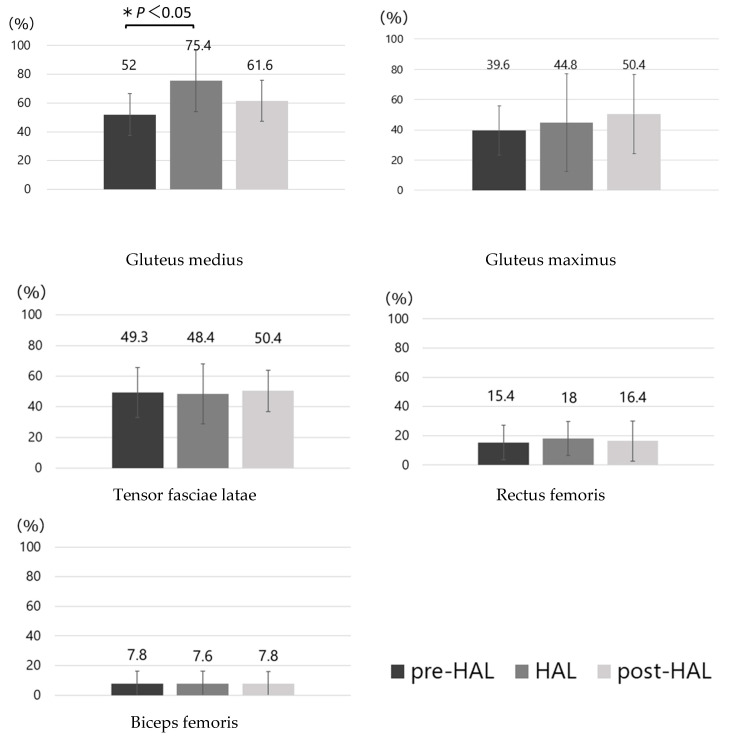
Mean muscle activity. Muscle activity levels normalized to maximum contraction are reported along the y-axis. Comparisons are made between the pre-HAL, HAL, and post-HAL conditions for each muscle. ANOVA was performed, followed by Bonferroni’s multiple comparison test for the three comparison patterns: pre-HAL vs. HAL, HAL vs. post-HAL, and pre-HAL vs. post-HAL. A significant difference is found between pre-HAL and post-HAL for the gluteus medius (*: *p* < 0.05). HAL, hybrid assistive limb robot; ANOVA, repeated-measures analysis of variance.

The %MVC values for each muscle in the pre-HAL, HAL, and post-HAL periods are shown in Figure 5. These showed a significant increase in mean gluteus medius activity (pre-HAL, 52 ± 14.6%; HAL, 75.4 ± 21.3%; *p* < 0.05). No significant differences were observed in the other muscles.

### 3.4. Motion Analysis

**Figure 6:**
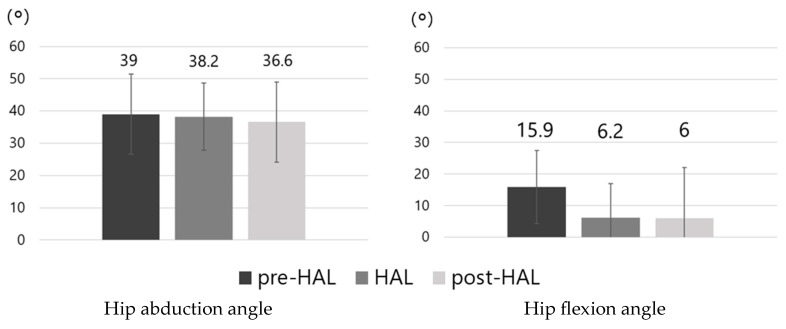
Mean ROM of hip abduction and flexion. No significant differences are observed in either abduction or flexion angles. ROM, range of motion.

The results of hip abduction and flexion angles during hip abduction–adduction movements in the pre-HAL, HAL, and post-HAL periods are shown in Figure 6. Although no significant differences were observed, the hip flexion angle tended to decrease in the HAL (6.2 ± 10.8°) and post-HAL (6 ± 16.1°) periods compared with that in the pre-HAL period (15.9 ± 11.5°).

### 3.5. Safety

**Figure 7:**
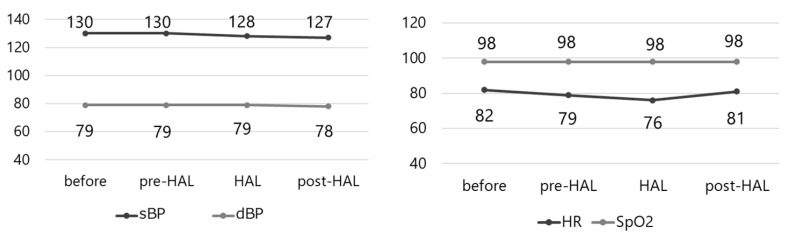
Vital signs (blood pressure, heart rate, and SpO2). No variations are observed in vital signs. sBP, systolic blood pressure; dBP, diastolic blood pressure; HR, heart rate; SpO2, percutaneous oxygen saturation.

Figure 7 shows the variations in the vital signs measured before and after the pre-HAL, HAL, and post-HAL sessions. No significant changes were observed in the vital signs. The VAS score for hip pain was 0 for all the participants, and none reported experiencing pain after the exercise.

## 4. Discussion

The single-joint HAL was developed to support the movement of the knee and elbow joints. In this study, we utilized a tripod-based platform with modified attachments, previously developed for the shoulder HAL [12], to create a “hip abduction HAL” capable of assisting hip abduction–adduction movements. We then evaluated muscle activity and safety during its use in healthy participants.

In terms of muscle activity, the results showed that using the hip abduction HAL significantly increased %MVC only in the gluteus medius. No significant changes were observed in the other muscles, and the %MVC of the tensor fascia latae, which also contributes to hip abduction, remained stable. In previous studies investigating HAL, it has been reported that HAL assistance can reduce the activity of compensatory muscles and relatively increase the activity of the primary movers [15]. Among patients with hip dysfunction, including those with osteoarthritis, the gluteus medius—primarily responsible for abduction—is often more atrophied compared to the tensor fasciae latae, which primarily contributes to hip flexion [16]. Consequently, it is thought that these patients perform an abduction movement that relies on compensatory hip flexion by the tensor fascia latae rather than true abduction through the gluteus medius. Furthermore, in patients with hip osteoarthritis, weakness or dysfunction of the gluteus medius contributes to gait abnormalities. Furthermore, during walking, there is a muscle imbalance characterized by overactivity of the tensor fasciae latae relative to the gluteus medius [17,18]. Based on these findings, it is not sufficient to strengthen the gluteus medius during postoperative rehabilitation after THA; rather, it is necessary to suppress the excessive activity of the tensor fasciae latae while targeting the gluteus medius. One possible reason that there was no significant change in the tensor fasciae latae %MVC in this study is that the hip abduction HAL specifically assists the gluteus medius, the primary muscle involved in hip abduction, thereby allowing the hip abduction movement to occur while relatively suppressing compensatory activation of the tensor fasciae latae. Furthermore, the fact that no significant differences were observed in other muscle groups suggests that the hip abduction HAL focuses primarily on hip abduction–adduction without influencing movements such as hip flexion/extension or knee flexion/extension. Moreover, from the perspective of muscle activation, these observations suggest that performing hip abduction from a neutral to a slightly extended position is crucial for effectively engaging the gluteus medius.

Our motion analysis results showed no significant differences in hip abduction or flexion angles during the hip abduction–adduction exercises. However, when using HAL, we observed a reduction in the hip flexion angle. Additionally, during HAL-assisted trials, the %MVC of the gluteus maximus increased and that of the tensor fasciae latae remained approximately the same as during pre-HAL. This suggests that the HAL device helps to mitigate excessive hip flexion during hip abduction, thereby enabling exercises to be performed in a more appropriate limb position. Moreover, although the changes did not reach statistical significance, after the participants used the HAL, subsequent hip abduction–adduction exercises performed without the HAL showed an increased %MVC of the gluteus medius and gluteus maximus, with the tensor fasciae latae remaining essentially unchanged and the hip flexion angles decreased. This implies that using the HAL may facilitate a learning effect, enabling the participants to perform hip abduction in a more optimal position, even without the device. This is an important factor in improving muscle balance and efficiently activating the hip abductors, particularly the gluteus medius.

Previous studies on single-joint HAL systems modified for shoulder and ankle joints demonstrated their safety during joint movements [12,13]. In the present study, no adverse events were observed in terms of vital signs or VAS scores, confirming the safety of hip abduction–adduction exercises performed with the hip abduction HAL.

Overall, this study showed that the hip abduction HAL enables efficient and safe gluteus medius-focused hip abduction exercises. This suggests that the hip abduction HAL could serve as a promising tool for improving gait and rehabilitation of the perihip muscles in patients with hip osteoarthritis.

This study had several limitations. A primary limitation of this study is that the participants were healthy individuals rather than patients with hip osteoarthritis. Such patients may present with pathological factors such as restricted range of motion and marked muscle weakness, leaving uncertainty as to whether the same efficacy and safety observed here would apply. Hence, clinical application must be approached cautiously, taking into account each patient’s pathological state and individual physical characteristics. Additionally, this study evaluated muscle activity and motion analysis only during a single session of hip abduction exercise. We did not assess long-term outcomes or the persistence of motor learning effects. In previous research, long-term gait training with a bilateral lower-limb HAL in patients with progressive neuromuscular degenerative diseases, such as spinal and bulbar muscular atrophy, has been reported to maintain walking ability for up to five years [19]. However, within the scope of our literature review, no studies were found that investigated the long-term use or long-term effects of single-joint HAL devices, such as the one used in this study. Consequently, further studies involving patients with hip osteoarthritis are needed to elucidate how the prolonged use of the hip abduction HAL may influence changes in movement patterns and the stabilization of muscle activity, as well as whether any benefits persist after the device is no longer in use.

In the future, it will be essential to investigate the feasibility of hip abduction HAL use in patients who have undergone THA for hip osteoarthritis and to conduct long-term clinical studies to confirm the effectiveness of the hip abduction HAL in improving gait patterns over time.

## 5. Conclusions

The HAL effectively promoted gluteus medius muscle activity and ensured its safe use in healthy adults. Future studies should evaluate the long-term effects and clinical applicability of THA. Demonstrating the efficacy of the hip abduction HAL as a promising tool for periarticular hip muscle rehabilitation is essential.

## Data Availability

The data are available from the corresponding author upon reasonable request.

## Abbreviations

The following abbreviations are used in this manuscript:

ADL	activities of daily living
EMG	electromyography
HAL	hybrid assistive limb
MVC	maximum voluntary contraction
THA	total hip arthroplasty
VAS	visual analog scale
